# Regulation of G0/G1 Switch Gene 2 (G0S2) Protein Ubiquitination and Stability by Triglyceride Accumulation and ATGL Interaction

**DOI:** 10.1371/journal.pone.0156742

**Published:** 2016-06-01

**Authors:** Bradlee L. Heckmann, Xiaodong Zhang, Alicia M. Saarinen, Jun Liu

**Affiliations:** 1 Department of Biochemistry & Molecular Biology, Mayo Clinic College of Medicine, Scottsdale, Arizona, United States of America; 2 Metabolic HEALth Program, Mayo Clinic, Scottsdale, Arizona, United States of America; 3 Mayo Graduate School, Rochester, Minnesota, United States of America; University of Graz, AUSTRIA

## Abstract

Intracellular triglyceride (TG) hydrolysis or lipolysis is catalyzed by the key intracellular triglyceride hydrolase, adipose triglyceride lipase (ATGL). The G0/G1 Switch Gene 2 (G0S2) was recently identified as the major selective inhibitor of ATGL and its hydrolase function. Since G0S2 levels are dynamically linked and rapidly responsive to nutrient status or metabolic requirements, the identification of its regulation at the protein level is of significant value. Earlier evidence from our laboratory demonstrated that G0S2 is a short-lived protein degraded through the proteasomal pathway. However, little is currently known regarding the underlying mechanisms. In the current study we find that 1) protein degradation is initiated by K48-linked polyubiquitination of the lysine- 25 in G0S2; and 2) G0S2 protein is stabilized in response to ATGL expression and TG accumulation. Mutation of lysine-25 of G0S2 abolished ubiquitination and increased protein stability. More importantly, G0S2 was stabilized via different mechanisms in the presence of ATGL vs. in response to fatty acid (FA)-induced TG accumulation. Furthermore, G0S2 protein but not mRNA levels were reduced in the adipose tissue of ATGL-deficient mice, corroborating the involvement of ATGL in the stabilization of G0S2. Taken together our data illustrate for the first time a crucial multifaceted mechanism for the stabilization of G0S2 at the protein level.

## Introduction

The process of lipid catabolism, also known as lipolysis in adipose tissue, is a mechanism that plays a central role in governing global lipid and energy homeostasis. This catabolic pathway in which fatty acids (FA) and glycerol are liberated from neutral triglyceride (TG) stores is essential for adaptive energy response [1]. FAs released into circulation from adipose lipolysis are used for a variety of cellular processes including as energy substrates in the liver [2]. Lipolysis in adipose tissue has been shown to be rapidly responsive to nutrient status [1,3]. Fasting and feeding can alter lipolytic output in an acute manner in response to β-adrenergic and insulin signaling, respectively.

Over the last decade the field of lipolysis has moved forward dramatically owing to the discovery of adipose triglyceride lipase (ATGL) [4–6]. Multiple studies utilizing cell and knockout mouse models have since established ATGL as the rate-limiting enzyme regulating lipolysis in both adipose and non-adipose tissues [5,7–13]. Recently, work by our laboratory identified a small basic protein encoded by G0/G1 Switch Gene 2 (G0S2) as a potent endogenous inhibitor of ATGL [14]. Direct binding between the hydrophobic domain of G0S2 and the patatin-like domain of ATGL was shown to convey inhibition of ATGL’s hydrolase activity *in vitro* and ATGL-mediated lipolysis in various cell types [14–16]. Further studies in mice have demonstrated the *in vivo* role of G0S2 as a major metabolic and energy regulator through its inhibitory action on ATGL [15,17–20]. As with lipolytic activation, G0S2 gene expression was found to be closely related to nutrient status and easily fluctuates depending on exogenous stimuli [14,15]. A separate study of ours has shown that G0S2 is a highly unstable protein that is degraded via the ubiquitin-proteasome pathway [12]. We speculate that the ability to rapidly turnover G0S2 protein allows cells to adjust lipolytic rates swiftly in response to changes in nutritional and hormonal signals.

To date, little is known regarding the specific mechanisms that govern G0S2 protein turnover and the regulation of G0S2 protein expression as a whole. In the present study, we have identified a potential ubiquitination site of G0S2, and obtained novel evidence detailing the involvement of FAs and ATGL in the control of G0S2 protein stability.

## Materials and Methods

### Animal Study and Ethics Statement

Global ATGL knockout (ATGL KO) mice (Courtesy of Rudolph Zechner, University of Graz) and wild type (Wt) C57BL/6J littermates were maintained in the animal facility at Mayo Clinic Arizona. Animal care and procedures were in accordance with the guidelines and regulations of the Mayo Clinic Institutional Animal Care and Use Committee, which approved this study (protocol number: A17813). All mice were given free access to water and were fed a standard chow diet (Test Diet, %5001, 10% calories as fat). For experiments, female mice at 8-weeks of age were used unless otherwise indicated. All sacrifice was performed under isofluran anesthesia, and all efforts were made to minimize suffering.

### Antibodies and Reagents

G0S2 antibody was produced by our laboratory as described previously [14]. Antibodies against HA-tag and murine ATGL were purchased from Cell Signaling. FLAG antibody and anti-FLAG conjugated protein A/G beads were purchased from Sigma-Aldrich. Anti-βactin antibody was used for all loading controls and purchased from Sigma-Aldrich. Anti-CGI58/Abhd5 antibody was purchase from ProteinTech. All compounds used for 3T3-L1 differentiation including isoproterenol (ISO), 3-isobutyl-1-methylxanthine (IBMX), and dexamethasone were purchased from Sigma-Aldrich. MG132, cyclohexamide (CHX), and T863 were purchased from Sigma-Aldrich and used at the indicated concentrations.

### Cell Culture

3T3-L1 fibroblasts were maintained in high glucose Dulbecco’s modified Eagle’s medium (DMEM) (Invitrogen) containing 10% newborn calf serum (Lonza), 1% penicillin/streptomycin (Invitrogen), and 1% glutamine (Invitrogen). For differentiation into adipocytes, 3T3-L1 fibroblasts were switched to media as described above, containing 10% fetal bovine serum (Sigma) in liu of newborn calf serum. In addition this media also contained 1ug/ml insulin, 0.25 uM dexamethasone, and 0.5 mM IBMX. Following three days of treatment, dexamethasone and IBMX were removed from the culture media. Following two days of insulin only treatment, cells were maintained in DMEM containing 10% fetal bovine serum, 1% penicillin/streptomycin, and 1% glutamine. HeLa cells and G0S2 stable HeLa cells were cultured in DMEM containing 10% heat-inactivated fetal bovine serum (Invitrogen) with 1% penicillin/streptomycin and 1% glutamine. All cells were kept at 37°C in a 5% CO_2_ atmosphere.

### Free fatty acid (FFA) treatment

Sodium Oleate (OA) (Sigma-Aldrich) was prepared and conjugated to bovine serum albumin (BSA) at a molar ratio of 6:1 (OA:BSA). A solution of fatty acid free BSA (Sigma-Aldrich) in DMEM was used. Upon addition of OA, conjugation was accomplished by heating at 65°C for 5 min. The BSA conjugated OA solution was used at a final concentration of 400uM unless otherwise noted.

### Gene expression and siRNA mediated gene knockdown

Transient expression of G0S2, ATGL, and Ubiquitin were accomplished utilizing Lipofectamine 2000 (Invitrogen) according to the manufacturer’s instructions. Eukaryotic expression vectors for FLAG-G0S2, non-tagged pRK-G0S2, pRK-ATGL and pRK-CGI-58 were cloned as reported previously [14,21]. HA-tagged ubiquitin and mutant K48R ubiquitin were purchased from Addgene. Both pRK-G0S2 and FLAG-G0S2 were used as templates to construct the lysine mutants of G0S2 using a site-directed mutagenesis kit per the manufacturer’s instructions (Agilent). The primer sets are as follows; K12R FWD: 5’—ATCCCTCTGGCCAGGGAGATGATGGC—3’ and REV: 5’—GCCATCATCTCCCTGGCCAGAGGGAT—3’, K18R FWD: 5’—ATGGCGCAGCGGCCCCGAGGGAAGCTAGTG—3’ and REV: 5’—CCCTCGGGGCCG CTGCGCCATCATCTCCTT—3’, K22R FWD: 5’—CCCCGAGGGCGGCTAGTGAAGCTA TACGTG—3’ and REV: 5’—CTTCACTAGCCG CCCTCGGGGCTTCTGCGC– 3’, K25R FWD: 5’—AAGCTAGTGCGGCTATACGTGCTGGGCAGT– 3’ and REV: 5’—CACGTA TAGCCGCACTAGCTTCCCTCGGGG—3’, K77/85R FWD: 5’—CCACAGGCAGGCCCTGCTGGCAGGAGGCAAGGCACAGGAGGCGACCCTGTGCAGCCGGGCCCTGTCCCTCCGGCAGCACGCCTCT -3’ and REV: 5’—AGAGGCGTGCTGCCGGAGGGACAGGGCCCGGCTGCACAGGGTCGCCTCCTGTGCCTTGCCTCCTGCCAGCAGGGCCTGCCTGTGG—3’.

Gene knockdown was achieved using Lipofectamine RNAi Max transfection reagent (Invitrogen) according to the manufacturer’s instructions. Stealth siRNA was purchased from Invitrogen. For experimental ATGL knockdown, two siRNA’s were combined for maximum knockdown efficiency. The sequences for the first mATGL siRNA are as follows; Sense: 5’- UCA GAC GGA GAG AAC GUC AUC AUA U—3’ and Anti-sense: 3’—AUA UGA UGA CGU UCU CUC CGU CUG A—5’. The second mATGL siRNA was composed of Sense: 5’—CCA GGC CAA UGU CUG CAG CAC AUU U– 3’ and Anti-sense: 3’—AAA UGU GCU GCA GAC AUU GGC CUG G—5’. Stealth RNAi Negative control MED GC (Invitrogen #10620312) was used for all control knockdowns. Please note this was specially order in lyophilized form instead of being pre-diluted.

### Cell lysis, immunoblotting and immunoprecipitation

For immunoblotting analysis, 3T3-L1 adipocytes, HeLa cells, G0S2 stably expressing HeLa cells, or adipose tissue samples were lysed in a buffer containing 50 mM Tris-HCl (pH 8.0), 135 mM NaCl, 10 mM NaF, 1% NP-40, 0.1% SDS, 0.5% sodium deoxycholate, 1.0 mM EDTA, 5% glycerol, and 1 × Complete protease inhibitor mixture (Roche Applied Science). The lysates were clarified by centrifugation at 20,000 × g for 10 min and then mixed with an equal volume of 2x SDS sample buffer. Equivalent amounts of protein were resolved using 1D SDS-PAGE, followed by transfer to nitrocellulose membranes. Individual proteins were blotted with primary antibodies at appropriate dilutions. Peroxide-conjugated secondary antibodies were incubated with the membrane at a dilution of 1:5000. The signals were then visualized by chemiluminescence (Supersignal ECL, Pierce) using an ImageQuant LAS4000 imaging system (GE HealthCare Life Sciences). For immunoprecipitation, cells were lysed in a buffer containing 50mM Tris-HCl (pH 8.0), 135mM NaCl, 10mM NaF, 1% NP-40, 1.0mM EDTA, 5% glycerol and protease inhibitors. Following clarification, lysates were mixed with the appropriate amount of anti-FLAG beads (Sigma-Aldrich) for 1h at 4°C. The beads were subsequently washed 6x in lysis buffer and were then mixed with 1x SDS sample buffer and the proteins were detected by immunoblotting analysis.

### RNA Extraction and Real-Time PCR

Total RNA was isolated from mouse adipose tissue samples using the RNeasy Plus Mini Kit (Qiagen) according to the manufacturer's instructions. cDNA was synthesized from total RNA by LongRange reverse transcriptase (Qiagen) with Oligo d(T). The resulting cDNA was subjected to real-time PCR analysis with SYBRGreen PCR Master Mix (Invitrogen) on an Applied Biosystems 7900 HT Real-Time PCR System. Data was analyzed using the comparative cycle threshold (ΔΔCt) method. The mRNA levels of genes normalized to β-actin were presented as relative to the Wt control. qPCR primers for β-actin, ATGL and G0S2 were used as previously published [18]. PCR product specificity was verified by postamplification melting curve analysis and by running products on an agarose gel.

### Intracellular Triglyceride Assay

Total intracellular triglycerides were measured at the basal state from HeLa and G0S2 stable HeLa cells. The Infinity Triglyceride Assay kit (Thermo Scientific) was used according to the manufacturer’s protocol.

### Statistical Analysis

Data are shown as mean ± S.D. Statistical significance was determined by the Student's unpaired t test (two-tailed). Group differences considered significant for p < 0.05

## Results

### G0S2 is a short-lived protein targeted for proteasomal degradation by polyubiquitination

**Fig 1:**
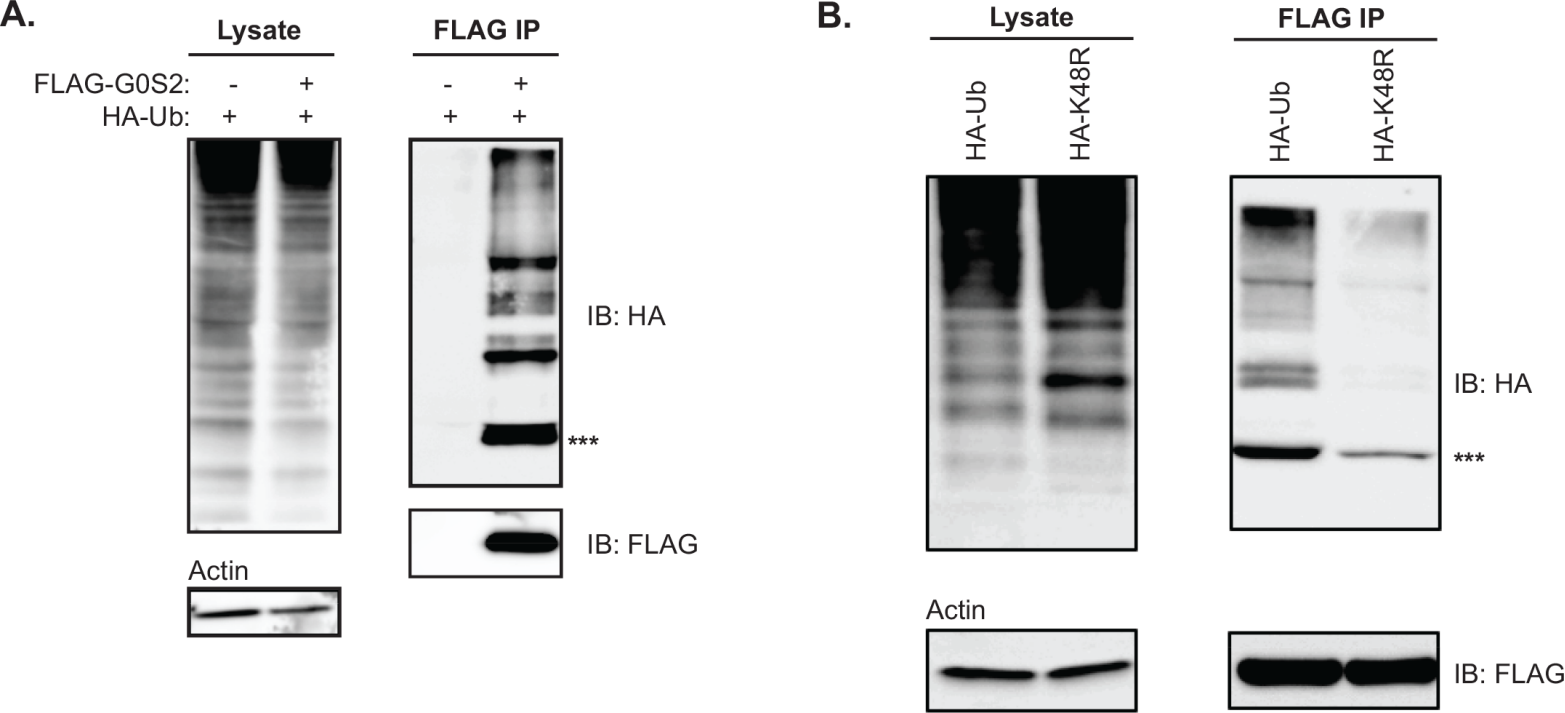
Post-translational modification of G0S2 by poly-ubiquitination. (A) HeLa cells were co-transfected with HA-tagged ubiquitin (Ub) and FLAG-G0S2. Following immunoprecipitation (IP) for FLAG, Ub-binding was assayed by immunoblot for HA-tag. Both lysate and IP blots are shown. (B) HeLa cells were co-transfected as described above, however HA-tagged K48R mutant ubiquitin was included alongside wild-type HA-Ub. Lysate and IP immunoblots are shown. ^***^ Represents mono-ubiquitinated G0S2. All data are representative of 3 independent experiments, performed in triplicate.

Previous evidence from our laboratory has demonstrated that G0S2 protein is rapidly degraded through the proteasomal pathway [12]. The most established mechanism targeting proteins for proteasomal degradation is polyubiquitination. To determine if G0S2 would be a substrate for polyubiquitination, we transiently co-expressed HA-tagged ubiquitin with or without FLAG-tagged G0S2 in HeLa cells. Immunoprecipitation followed by immunoblotting analysis revealed multiple HA-tagged protein bands specifically bound to anti-FLAG antibody (Fig 1A). To confirm that the smeared banding pattern is caused by mono- and polyubiquitination of FLAG-G0S2, we repeated our co-expression assay using a HA-tagged K48R ubiquitin mutant. Polyubiquitination through lysine residue at position 48 (K48) of ubiquitin is known to be the most potent signal for targeting substrate proteins to the proteasome [22,23]. The K48R mutant lacks the ability to form K48-linked polyubiquitin chains as reported previously [22]. Compared with that of wild type ubiquitin, co-expression of FLAG-G0S2 with the HA-ubiquitin/K48R mutant resulted in an approximate 90% reduction in the total HA signal in the anti-FLAG immunoprecipitates (Fig 1B). While bands of higher molecular weight representing polyubiquitinated G0S2 were almost completely lost, a significant though decreased amount of the mono-ubiquitinated form was still detectable. Taken together, these findings demonstrate that G0S2 can be both mono- and polyubiquitinated, and the majority of ubiquitination observed is polyubiquitination mediated through K48 of the ubiquitin protein.

### Mutation of lysine-25 (K25) of G0S2 decreases ubiquitination and increased protein stability

**Fig 2:**
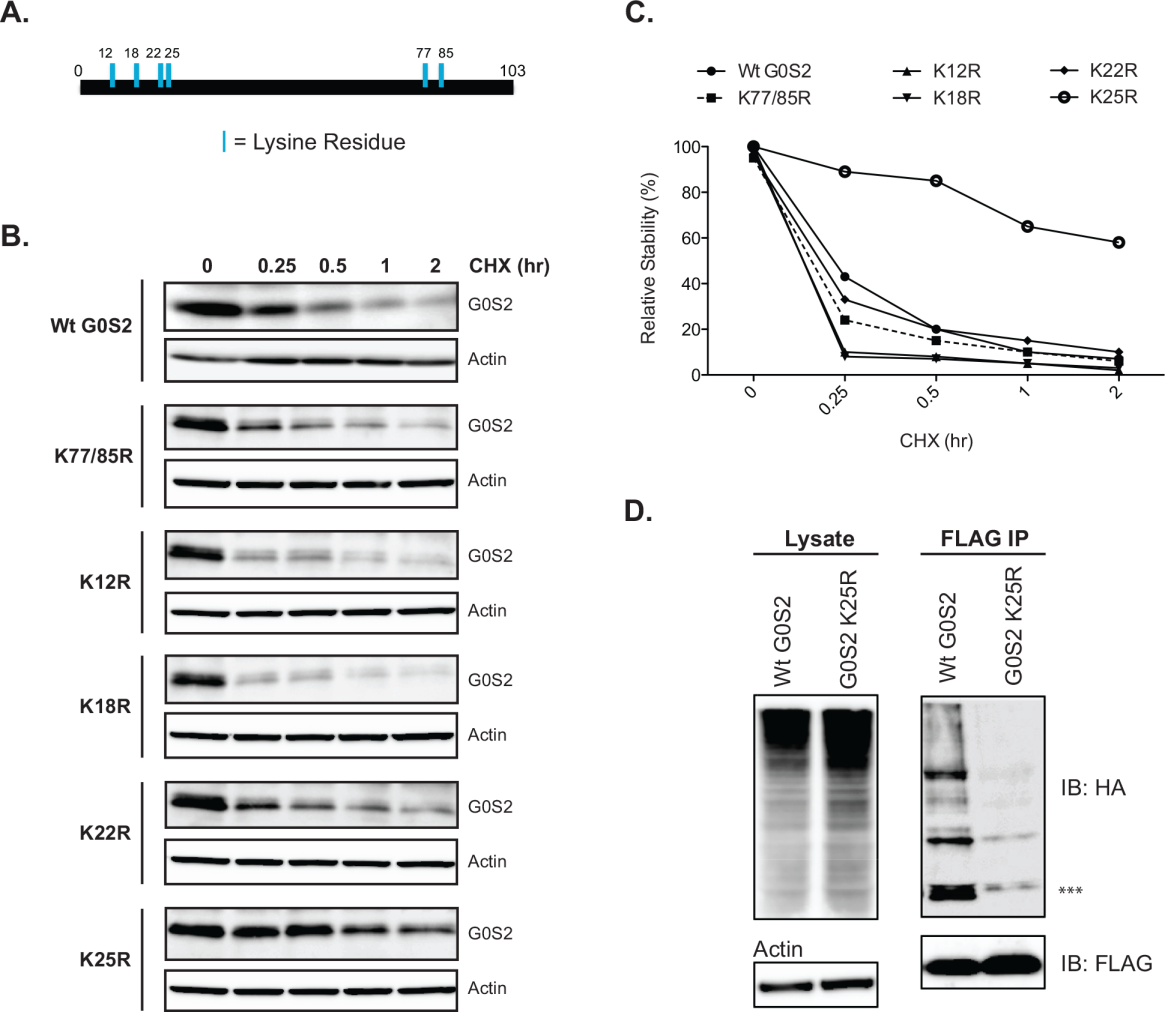
Ubiquitin attachment to G0S2 occurs on the lysine residue at position 25. Individual lysine mutants of G0S2 were constructed by site directed mutagenesis. (A) Illustration of the 6 conserved lysine residues present in G0S2. (B) Lysine mutants of G0S2 were expressed in HeLa cells and then treated with 10uM CHX as indicated. Immunoblot analysis for G0S2 expression is shown. (C) Relative stability quantification of various G0S2 lysine mutants in response to CHX treatment. (D) The K25R mutant of G0S2 was co-expressed with HA-Ub in HeLa cells; IP was completed as described in Fig 1. Immunoblot analysis of both total lysate and IP are shown. ^***^ Represents mono-ubiquitinated G0S2. All data are representative of 3 independent experiments, performed in duplicate (B,C) and triplicate (D).

The canonical pathway for ubiquitin modification of substrate proteins is the binding of ubiquitin to a lysine residue of a target protein. There are 6 conserved lysine residues found in the G0S2 protein, 4 of which are clustered at the N-terminal region and 2 close to the C-terminus (Fig 2A). To determine which lysine residue(s) are obligatory for ubiquitin binding, we created a series of point mutants by site-directed mutagenesis. To ensure conservation of charge, we substituted lysine (K) for arginine (R). Each G0S2 mutant was individually expressed in HeLa cells and a 2-h CHX treatment was performed to determine the impact of each K→R substitution on protein stability. As shown in Fig 2B, the only mutation that improved G0S2 stability when compared to wild-type G0S2 was the K25R mutant. When quantified, the K25R mutant had an approximate 45–60% higher relative stability across all time points through the 2-h time period than the wild-type protein and the remaining mutants (Fig 2C). Using our co-expression system detailed above in Fig 1, we found that the G0S2 K25R mutant had a marked reduction in ubiquitination when compared to the wild-type protein (Fig 2D). Together, these results indicate that lysine-25 of G0S2 is the predominant site of ubiquitin attachment. Loss of lysine-25 not only reduces the overall level of ubiquitination, but as a result improves the protein stability of G0S2.

### G0S2 is stabilized in response to TG accumulation

**Fig 3:**
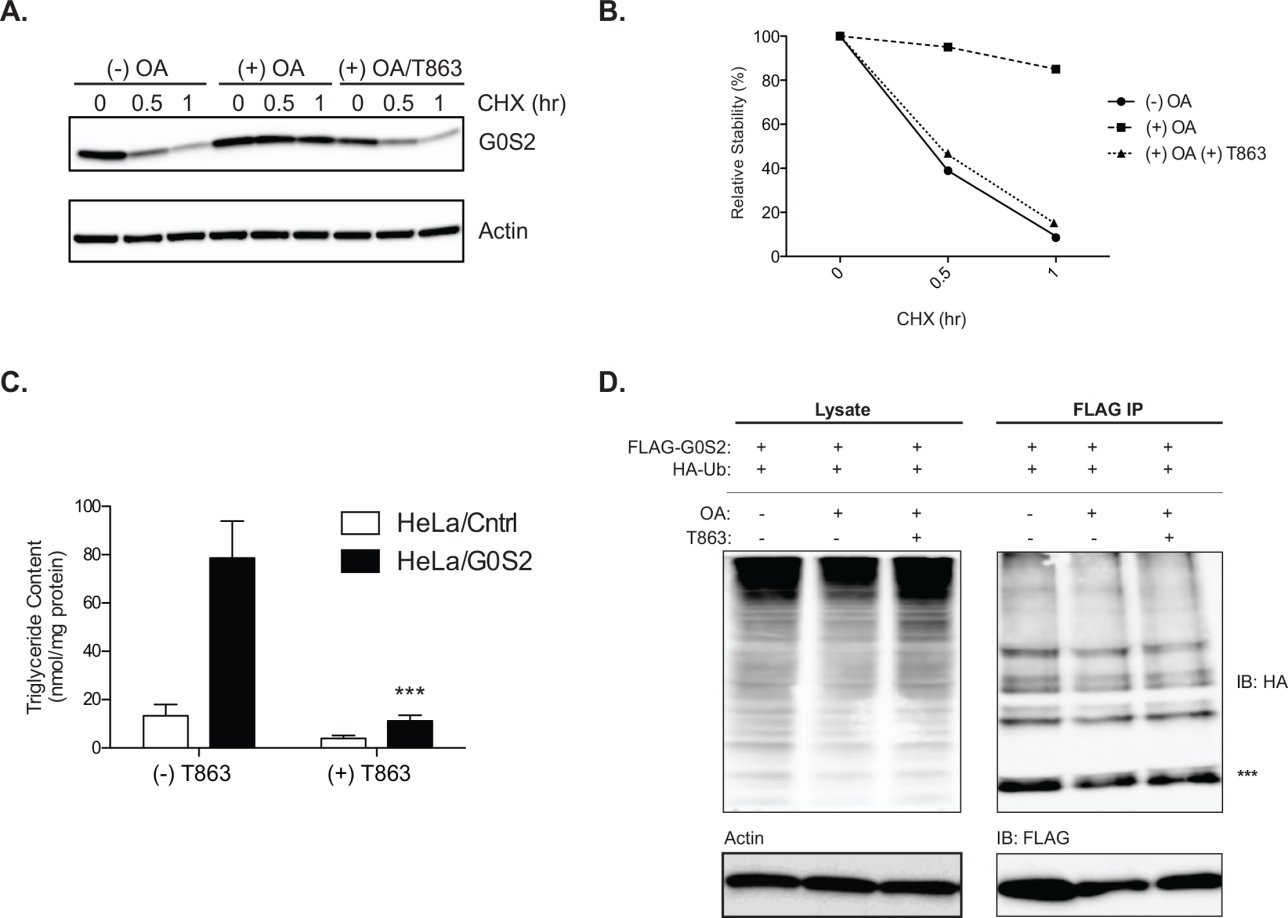
Fatty acids and triglyceride accumulation improves G0S2 stability but does not effect ubiquitination. (A) HeLa/G0S2 cells were loaded with 400uM oleic acid (OA) for 8 hours in the presence or absence of 10uM T863. Following loading, cells were treated with 10uM CHX as indicated. G0S2 expression was measured by immunoblot analysis and (B) relative quantification of G0S2 stability was determined. (C) Basal TG content was determined in HeLa/Cntrl cells and HeLa/G0S2 cells in the presence or absence of 10uM T863. (D) HeLa cells were transfected as described in Fig 1 with FLAG-G0S2 and HA-Ub. Cells were treated with 400uM OA and 10uM T863 as listed prior to IP for FLAG. Lysate and IP immunoblots are shown. *** Represents mono-ubiquitinated G0S2. All data are representative of three independent experiments, performed in duplicate for B-D. For TG content assay (C) (*n =* 3 per group, performed in triplicate) ***p <0.*001*.

G0S2 expression is known to be elevated in response to increased influx of FAs in both adipose tissue and liver [15,18,24]. To test whether FAs might function to promote G0S2 protein stability, we treated HeLa cells stably expressing G0S2 (HeLa/G0S2) with exogenous oleic acid (OA) for 8-h prior to the CHX addition. As shown in Fig 3A, G0S2 protein was stabilized by OA at both 0.5- and 1-h time points of the CHX treatment (Fig 3A and 3B). Since G0S2 is a LD-localized protein, we next asked whether the stabilizing effect of OA is contingent upon the increased formation of TG-LDs. Diacylglycerol acyltransferases (DGATs) are a family of enzymes that govern the final committed step of TG synthesis, converting diacylglycerol and fatty acyl-CoA to TG. The DGAT inhibitor T863 has been shown to potently decrease FA incorporation into TG and LD accumulation in various cells and primary tissues [25]. In accordance to what was observed previously, HeLa/G0S2 cells exhibited an approximate 3-fold increase in the intracellular TG content when compared to the control cells upon OA treatment [14]. Pretreatment of cells with T863 prior to OA drastically reduced the TG content in both control and HeLa/G0S2 cells (Fig 3C). Interestingly, T863 largely abolished the stabilizing effect of OA on G0S2 protein in HeLa/G0S2 cells (Fig 3A and 3B). However, regardless of DGAT inhibition, OA treatment did not significantly affect the ubiquitination of FLAG-G0S2 by co-expressed HA-ubiquitin (Fig 3D). The results suggest that while G0S2 protein is less stable in the absence of LDs, intracellular FA and TG levels are not causatively related to G0S2 ubiquitination.

### G0S2 protein expression is diminished in adipose tissue of ATGL knockout mice

**Fig 4:**
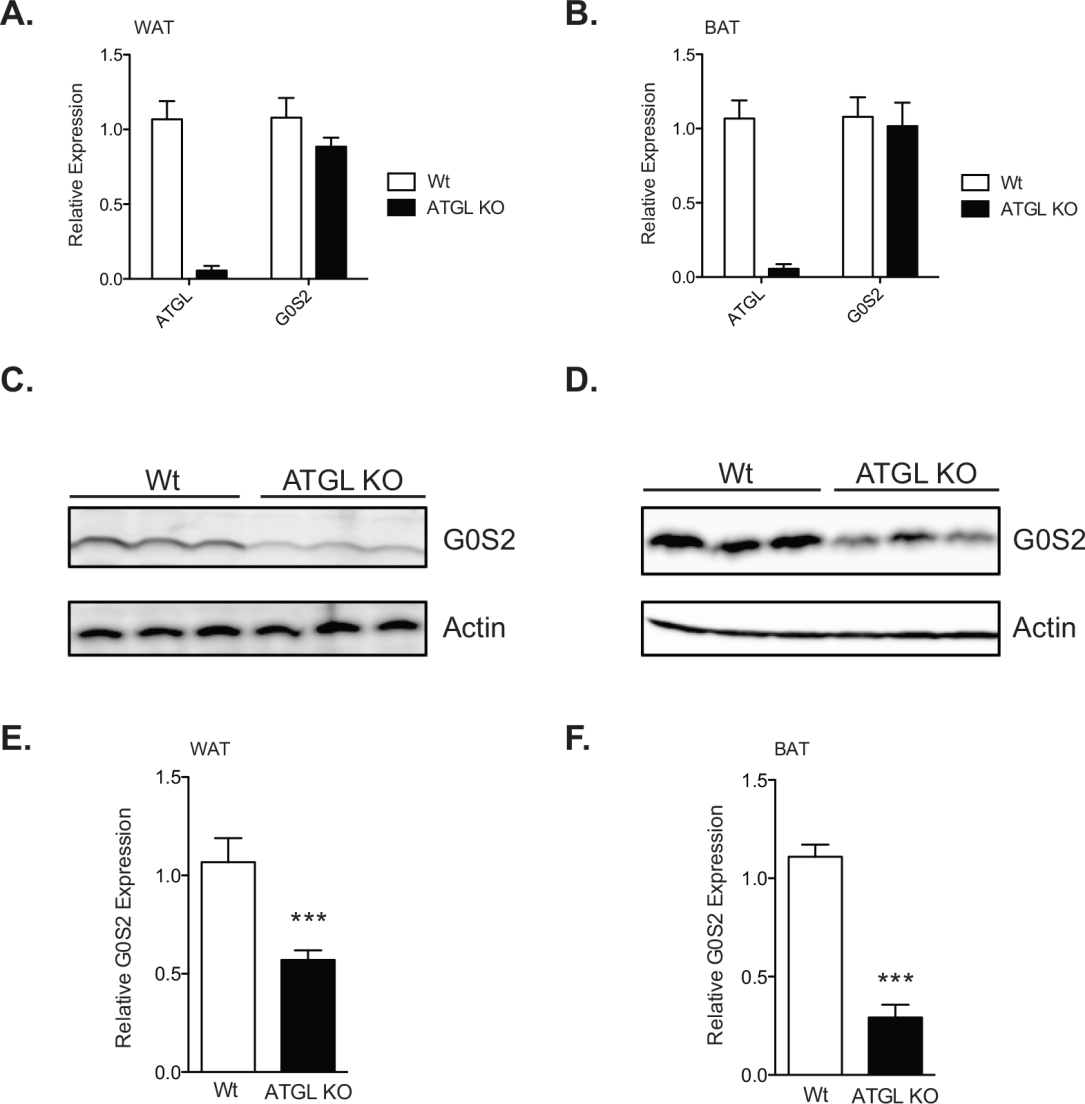
G0S2 stability is decreased in mice lacking ATGL. Gonadal WAT and BAT was isolated from 8-week old female ATGL KO mice or wild-type (Wt) littermates. G0S2 transcriptional activity was measured by qPCR in (A) WAT and (B) BAT. Immunoblot analysis of G0S2 expression in (C) WAT and (D) BAT was measured. Relative expression was then quantified for (E) WAT and (F) BAT. For qPCR experiments (*n =* 5 per group in triplicate). For immunoblot analysis (*n =* 3, representative of 8 per group) ***p <0.*001*.

G0S2 protein expression has been shown to fluctuate *in vivo* across tissues in response to various metabolic stimuli. It is well known that G0S2 and ATGL are intimately associated with each other in a stable complex [14]. To evaluate if G0S2 protein levels could be influenced by ATGL *in vivo*, we isolated brown adipose tissue (BAT) and gonadal white adipose tissue (WAT) from mice with global deletion of ATGL (ATGL KO) and their wild-type littermates. In both adipose depots, G0S2 mRNA expression was independent of ATGL expression as revealed by qPCR analysis (Fig 4A and 4B). Interestingly, at the protein level, G0S2 expression was reduced in both adipose depots of the ATGL KO mice (Fig 4C and 4D). Compared with that of the wild type mice, there was a 50% and 70% reduction in the G0S2 protein content in WAT and BAT of the ATGL KO mice, respectively (Fig 4E and 4F). These results provide evidence that ATGL is able to influence G0S2 levels post-transcriptionally.

### Stabilization of G0S2 by ATGL is accompanied by decreased ubiquitination and independent of TG accumulation

**Fig 5:**
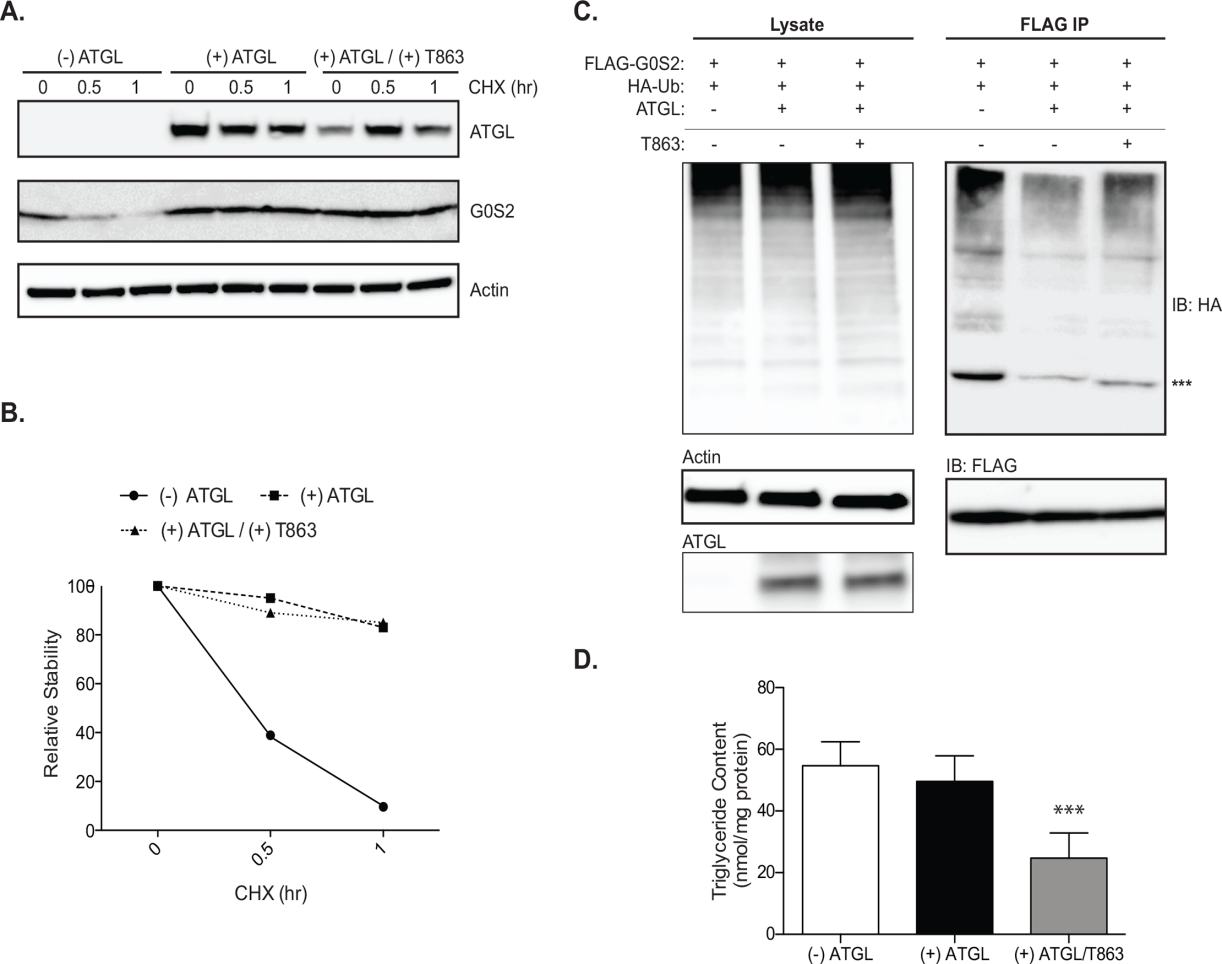
ATGL expression improves G0S2 stability by reducing ubiquitination. ATGL was expressed in HeLa/G0S2 cells by transient transfection. Cells were then pre-treated in the presence or absence of 10uM T863 for 4 hr and then 10uM CHX was added for a time course of 1 hr. (A) Immunoblot analysis of ATGL and G0S2 expression. (B) Quantification for relative stability is also shown. (C) Hela cells were transfected as previously described with FLAG-G0S2 and HA-Ub. Cells were treated for 4 hours in the presence or absence of 10uM T863 prior to FLAG IP. Lysate and IP immunoblots are shown. ^***^ Represents mono-ubiquitinated G0S2. (C) Basal TG content in HeLa/G0S2 cells expressing ATGL in the presence or absence of T863. (*** *p* < 0.001) All data are representative of three independent experiments, performed in duplicate.

To determine if ATGL could directly impact G0S2 protein stability, we transiently overexpressed ATGL in HeLa/G0S2 cells and monitored the levels of G0S protein over a 1-h period of CHX treatment. As shown in Fig 5A, ATGL expression led to stabilization of G0S2 protein throughout the duration of the treatment. While 70–75% of G0S2 protein was degraded at 30-min in cells transfected with vector alone, no significant decrease of G0S2 protein content was observed at the 30-min point when ATGL was overexpressed. G0S2 protein levels were approximately 9-fold higher in ATGL-overexpressing cells than in control cells consistently after 1 h of CHX treatment (Fig 5A and 5B). In the co-expression system, ATGL expression also caused a decreased ubiquitination of FLAG-G0S2 by HA-ubiquitin (Fig 5C). Interestingly, ATGL overexpression in HeLa/G0S2 cells did not significantly alter the intracellular TG content (Fig 5D). This is presumably due to the inhibition of ATGL by G0S2. However, compared to vehicle treated cells, cells treated with T863 exhibited a substantial reduction in TG content (Fig 5D). However, this did not alter the effect of ATGL expression on either G0S2 stability or its ubiquitination (Fig 5A–5C). These data suggest that ATGL stabilizes G0S2 *via* attenuating its ubiquitiantion and independently of TG accumulation in the cells.

### Activation by CGI-58 does not impact ATGL’s ability to stabilize G0S2

**Fig 6:**
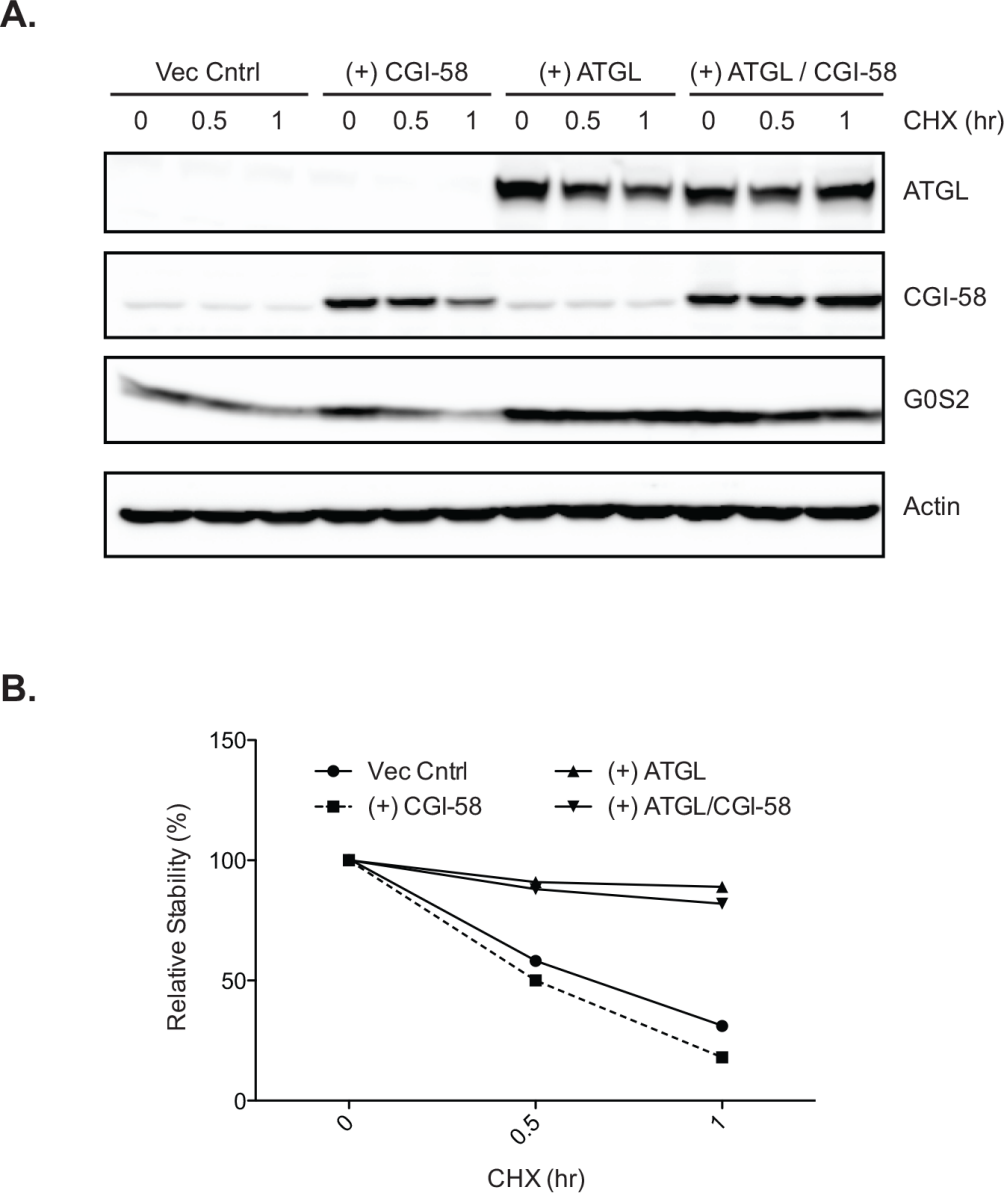
ATGL activation does not alter its ability to stabilize G0S2 protein. ATGL and CGI-58 were expressed singly or in combination in HeLa/G0S2 cells by transient transfection. Cells were then treated with vehicle alone or 10 μM of T863 for 4 h followed by 10 μM of CHX for a time course of 1 h. (A) Immunoblot analysis of ATGL, CGI-58 and G0S2 expression, and (B) quantification of relative stability are shown. All data are representative of three independent experiments performed in duplicate.

Since ATGL is coactivated by Comparative gene identification-58 (CGI-58), we also examined the effects of CGI-58 on G0S2 protein stability. To this end, we transiently co-expressed ATGL and CGI-58 in HeLa/G0S2 cells and then monitored levels of these proteins over a 1-h period of CHX treatment. Consistent with our earlier findings, expression of ATGL alone was able to stabilize G0S2 protein as shown by immunoblot analysis (Fig 6A) and relative stability quantification (Fig 6B). In contrast, single expression of CGI-58 had no impact on G0S2 protein turnover. Moreover, stabilization of G0S2 by ATGL was unaffected when CGI-58 was present (Fig 6A and 6B). These data demonstrate that activation by CGI-58 does not alter ATGL’s ability to stabilize G0S2 at the protein level.

## Discussion

G0S2 has been established as a key regulator of lipid metabolism by virtue of its ability to bind to and inhibit the hydrolase activity of ATGL. In the present study, we build on our previous observation that G0S2 is rapidly targeted for degradation by the proteasome [12], and have made the following novel observations; 1) G0S2 is targeted to the proteasome by canonical K48-linked polyubiquitination occurring at lysine-25; 2) TG accumulation stabilizes G0S2 without impact on its ubiquitination; 3) G0S2 protein expression is correlative to ATGL expression *in vivo;* and 4) ATGL overexpression decreases the ubiquitination and increases the stability of G0S2 protein regardless of the presence of the ATGL coactivator CGI-58.

Our evidence indicates both monoubiquitin and polyubiquitin attachment to G0S2 at lysine-25. We found that mutation of lyine-25 to arginine results in decreased ubiquitin binding and prominently increased protein stability. The polyubiquitination observed on G0S2 is K48-linked, a form widely-recognized as part of the predominant targeting mechanism for proteasomal degradation [22,23]. Another interesting finding is the reduction in monoubiquitination of G0S2 when we utilized the K48 ubiquitin mutant. This suggests that polyubiquitination of G0S2 may not stem solely from the elongation of bound mono-ubiquitin, but instead, result from the attachment of longer premade ubiquitin chains. In comparison to elongation, longer chain attachment was shown previously and may cause protein degradation in a more rapid manner [26–30], which is consistent with the short half-life and constitutive degradation that is observed with G0S2.

Since lipolysis is a vital component of the adaptive energy response system and whole body energy regulation, matching lipolysis to energetic demand is essential for global energy balance and lipid homeostasis. If G0S2 is rapidly degraded through ubiquitination as our data supports, a mechanism of stabilization may exist to increase G0S2 protein levels and thereby decrease lipolytic output when energy substrates are in excess. In this context, we discovered that elevated FA levels not only increase G0S2 gene expression [15] but also its protein stability *via* promoting formation of TG-lipid droplets (LD). FA and TG accumulation have been shown to contribute to the stabilization of a variety of LD coat proteins. For example, both fat specific protein 27 (FSP27) and perilipin 1 (Plin1) can be stabilized in response to enhanced TG accumulation in LDs [31,32]. It is possible that LD formation establishes a “scaffold” for the sequestration and thus protection of associated proteins such as FSP27, Plin1 and G0S2. As with FSP27 and Plin1, FAs fail to stabilize G0S2 protein if TG synthesis is abolished through inhibition of DGAT activity.

The most prominent biological property of G0S2 is its ability to interact with ATGL at high affinity. High affinity protein-protein interactions have been shown to increase protein stability at the LD surface. For instance, FSP27 exhibits an increased stability upon binding to heat shock cognate 70 (HSC70) [33]. Similarly, binding of Plin1 through the carboxy terminus of Plin1 results in stabilization of CGI-58 and retardation of its degradation in the proteasome [34]. Moreover, this interaction sequesters CGI-58 and prevents it from activating ATGL, thereby decreasing lipolytic capacity. We provide evidence that the G0S2 protein stability can be regulated by ATGL in a similar manner. We show that G0S2 stability is increased in the presence of ATGL regardless of CGI-58 when co-expressed in HeLa cells. This finding is consistent with the decreased levels of G0S2 protein but not of its mRNA in the adipose tissue of ATGL KO mice, and agrees with the previous evidence that CGI-58 does not impact ATGL-G0S2 binding [21]. As with the sequestration of CGI-58 by Plin1, the interaction of G0S2 with ATGL decreases lipolytic activation [14]. Both scenarios may contribute to low FA release from adipose tissue in the fed state.

While TG synthesis appears to elicit no effect on G0S2 ubiquitination, ATGL decreases G0S2 ubiquitination and mediates its stabilization independently of TG accumulation. In particular, we show that inhibition of TG synthesis by T863 results in decreased intracellular TG content and yet elicited no effect on ATGL’s ability to decrease G0S2 ubiquitination. It is tempting to speculate that ATGL binding may directly prevent the targeting of G0S2 by specific E3 ubiquitin ligase(s). While ATGL binding and TG accumulation seem to act independently to stabilize G0S2, we hypothesize that there is a cooperative functioning between these two mechanisms *in vivo*. Under conditions of heightened metabolic demand, G0S2 is constitutively degraded following synthesis at the endoplasmic reticulum, thereby favoring the ATGL-mediated TG mobilization. However, in the presence of influxed FAs, G0S2 protein may translocate to the newly formed LDs, where it forms stable complexes with ATGL. While the data presented here are supportive of our model demonstrating the stabilization of G0S2 at the protein level, separate mechanisms for expression regulation also exist. It is likely that a combination of transcriptional activation and increased protein stability occur *in vivo* to regulate the G0S2 protein levels.

In summary, the results from this study demonstrate a multifaceted regulation of G0S2 protein ubiquitination and stability. Since the role of G0S2 is of importance to lipid metabolism, the findings presented herein suggest that modulation of G0S2 protein stability could be a promising avenue for the manipulation of global energy homeostasis.
